# Aberrant Cerebral Activity in Early Postmenopausal Women: A Resting-State Functional Magnetic Resonance Imaging Study

**DOI:** 10.3389/fncel.2018.00454

**Published:** 2018-11-26

**Authors:** Si Zhang, Junhao Hu, Weijie Fan, Bo Liu, Li Wen, Guangxian Wang, Mingfu Gong, Chunyan Yang, Dong Zhang

**Affiliations:** Department of Radiology, Xinqiao Hosptial, Third Military Medical University, Chongqing, China

**Keywords:** early postmenopausal women, resting-state functional magnetic resonance imaging (rs-fMRI), degree centrality, functional connectivity, cognition, emotion

## Abstract

**Background**: Early postmenopausal women frequently suffer from cognitive impairments and emotional disorders, such as lack of attention, poor memory, deficits in executive function and depression. However, the underlying mechanisms of these impairments remain unclear.

**Method**: Forty-three early postmenopausal women and forty-four age-matched premenopausal controls underwent serum sex hormone analysis, neuropsychological testing and resting-state functional magnetic resonance imaging (rs-fMRI). Degree centrality (DC) analysis was performed to confirm the peak points of the functionally abnormal brain areas as the centers of the seeds. Subsequently, the functional connectivity (FC) between these abnormal seeds and other voxels across the whole brain was calculated. Finally, the sex hormone levels, neuroimaging indices and neuropsychological data were combined to detect potential correlations.

**Results**: Compared with the premenopausal controls, the early postmenopausal women exhibited significantly higher serum follicle-stimulating hormone (FSH) levels, more severe climacteric and depressive symptoms, worse sleep quality and more extensive cognitive impairments. Concurrently, the neuroimaging results showed elevated DC values in the left amygdala (AMYG.L), reduced DC values in the left middle occipital gyrus (MOG.L) and right middle occipital gyrus (MOG.R). When we used the AMYG.L as the seed point, FC with the left insula (INS.L), bilateral prefrontal cortex (PFC) and right superior frontal gyrus (SFG.R) was increased; these regions are related to depressive states, poor sleep quality and decreased executive function. When bilateral MOG were used as the seed points, FC with left inferior parietal gyrus (IPG.L), this area closely associated with impaired memory, was decreased.

**Conclusion**: These results illuminated the regional and network-level brain dysfunction in early postmenopausal women, which might provide information on the underlying mechanisms of the different cognitive impairments and emotional alterations observed in this group.

## Introduction

Menopause is a progressive recession of the female reproductive system and is characterized by a decline in ovarian function and the termination of menstruation, with excessive levels of follicle-stimulating hormone (FSH) and reduced levels of estrogen. Many menopausal women suffer from typical neuropsychiatric complaints, including anxiety, depression, doubtfulness and irritability (Studd, 2016), and more importantly, many of these women will face a high risk of long-term diseases featuring cognitive impairment, which may be a predictor of later development of Alzheimer’s disease (AD; Santoro et al., 2015). A rapidly expanding body of research now demonstrates that the estrogen play a central role in neuroprotection in the central nervous system (CNS), the state of estrogen deprivation could damage to neurons that modulate emotion and cognition. However, the potential mechanisms underlying this brain dysfunction in early postmenopausal women remain to be elucidated.

Recently, hormone therapy (HT) is commonly applied in the clinic and the researches are also conducted around it, and there are many longitudinal magnetic resonance imaging (MRI) studies but few cross-sectional MRI studies concerned postmenopausal women. Although longitudinal studies repeatedly demonstrated structural and functional brain abnormalities between the hormone-taking and placebo-taking postmenopausal women or in the same postmenopausal women before and after HT, the information from cross-sectional neuroimaging studies may also provide useful contributions to further clarify the correlation between cognitive impairments and brain dysfunction in early postmenopausal women. Cross-sectional MRI comparisons of early postmenopausal women and age-matched premenopausal women are of interest because the aging process of the brain also lead to structural and functional brain changes, and this was proved in the hypothesis of a limited window for the effects of HT (Georgakis et al., 2016; Aguirre-Vidal et al., 2017; Hwang et al., 2017). In these longitudinal studies, postmenopausal women who received HT had larger hippocampus/parahippocampal volumes and greater cognitive performance than those who received placebos (Eberling et al., 2003; Erickson et al., 2005; Boccardi et al., 2006; Lord et al., 2008). Girard et al. (2017) used task-based functional MRI (fMRI) to detect functional changes in the hippocampus, insula, occipital gyrus and prefrontal gyrus in postmenopausal women who received early HT compared to those who received placebos; these findings provided evidence for a beneficial effect of hormones on cognitive changes (Girard et al., 2017). Vega used resting-state fMRI (rs-fMRI) to find that early postmenopausal women with subjective cognitive impairment had concomitant changes in brain connectivity (Vega et al., 2016). Simultaneously, numerous studies have proved that the frontal cortex was involved in the executive function (Huang et al., 2015; Girard et al., 2017) and the insula was related to the negative emotional experiences and regulation of bodily homeostasis (von Leupoldt et al., 2008; Critchley, 2010), these functions of the two regions were associated with clinical symptoms among the early postmenopausal women. We designed this cross-sectional study to detect the brain dysfunction in early postmenopausal subjects.

In this study, resting-state fMRI was performed to reveal the spontaneous or intrinsic intra- and interregional connectivity in the brain for a further understanding of the brain networks in early postmenopausal women compared to age-matched control subjects. To detect alterations in regional cerebral activity, we used degree centrality (DC), a promising method, to calculate the number of correlations (or instantaneous functional connections) between cortical hubs and the rest of the regions of the brain (Buckner et al., 2009). The cortical hubs constitute the core architecture of the brain, which is consistent and stable in healthy subjects but highly vulnerable to pathological processes (Markett et al., 2016). To date, DC calculations have been widely applied to reveal the mechanisms of several cognition- or mood-related diseases, including hyperthyroidism (Li et al., 2017), schizophrenia (Palaniyappan and Liddle, 2014), AD (Guo et al., 2016), Parkinson’s disease (Peng et al., 2014) and depressive disorder (Shen et al., 2015). In contrast to other seed-selection approaches, which rely on a priori presumptions to select specific brain areas, DC can screen for regions with significant alterations in connectivity strength to be the impaired hubs (Tomasi and Volkow, 2011; Zhou et al., 2014). Subsequently, the peak points of those cortical hubs are used as the center of a sphere with a 6 mm radius, and these spheres are used in further seed-based functional connectivity (FC) analyses; the FC results between the seed nodes and the altered brain regions are used to unravel the potential mechanisms of the neurocognitive impairments. We expected that brain functional changes would be associated with the neuropsychological symptoms observed in early postmenopausal women.

## Materials and Methods

### Subjects

The local Medical Research Ethics Committee of Xinqiao Hospital (Chongqing, China) approved all the procedures, and written informed consent was obtained from all subjects before the study. Forty-three right-handed early postmenopausal women (age range: 45–50 years; mean age: 47.37 ± 1.59 years) were recruited by advertisements and selected according to the following two criteria (Berent-Spillson et al., 2012): (1) no menstruation during the past 12 months and FSH >40 IU/liter; and (2) typical clinical symptoms of menopause, such as night sweats and hot flashes. Forty-four right-handed, education-matched controls (age range: 45–50 years, mean age: 46.98 ± 1.61 years) who had regular menstrual cycles, FSH <11 IU/liter, and no menopausal symptoms were also recruited.

All participants were screened before the study for the following: general health; brain neuroanatomical abnormalities; acute psychiatric or behavioral disorders; a history of head injury, drug use or alcohol abuse; hysterectomy or bilateral oophorectomy; estrogen or progesterone use in the previous 6 months; and chronic illnesses, such as diabetes, hypertension, rheumatoid arthritis, cancer or other illnesses that may affect cognition. Additionally, participants with MR imaging contraindications or in-scanner head motion exceeding 1.5 mm or 1.5° were excluded.

### Experimental Design

All members of the two groups had their fasting serum FSH and estrogen levels assessed between 8 o’clock and 9 o’clock a.m. After serum collection, clinical symptoms and neuropsychological scale assessments were performed. Because some of the estrogen levels we detected in our hospital were outside of the reference range, the estrogen levels were excluded from the subsequent statistical analyses. After those assessments, MRI data were acquired over a 10-min resting-state block. Then, the subjects underwent cognitive testing in a quiet room. Each participant underwent all of these assessments in a single day. The data for the control subjects were collected in the follicular phase of the menstrual cycle, excluding the menstrual period (Aitken et al., 2008; Bixo et al., 2018).

### Neuropsychological Testing and Clinical Symptom Assessment

A series of related psychometric and clinical scales were completed by all of the subjects. The mood tests consisted of the Beck Depression Inventory-II (BDI-II, a 21-question multiple-choice self-report inventory that evaluates depression levels) and the Self-Rating Anxiety Scale (SAS, a 20-item self-report assessment designed to measure anxiety levels). Clinical symptoms were mainly measured by the Kupperman index (KI), which includes hot flashes, insomnia, anxiety, depression, dyspareunia, arthralgia, dizziness, weakness, headache, skin paresthesia, urinary symptoms and palpitations and weighs these factors to evaluate the severity of the climacteric symptoms, and the Pittsburgh Sleep Quality Index (PSQI) was used to assess the participants’ sleep quality.

### Image Acquisition

All subjects underwent rs-fMRI scanning on a 3.0T GE MRI system with a standard eight-channel phased-array head coil. Subjects were instructed to have no systematic cognitive or motor activity, to keep their eyes closed, and to avoid falling asleep during the scanning process. An echo-planar imaging sequence was used to collect the resting-state functional images; the parameters of this sequence were as follows: number of slices = 34; slice thickness = 5 mm, slice gap = 0 mm; TE = 30 ms, TR = 2300 ms, flip angle (FA) = 90°, field of view (FOV) = 240 × 240 mm^2^, matrix = 64 × 64, isotropic voxel size = 3 × 3 × 3 mm^3^ and number of time points = 270. Each participant underwent a resting-state block with duration of 621 s. A three-dimensional fast spoiled gradient-echo (3D FSPGR) sequence was used to obtain high-resolution brain structural images; the parameters of this sequence were as follows: number of slices = 124; slice thickness = 1.6 mm, slice gap = 0 mm; TE = 2.8 ms, TR = 450 ms, FA = 15°, FOV = 240 × 240 mm^2^, matrix = 256 × 256 and isotropic voxel size = 1.6 × 1.6 × 1.6 mm^3^.

### Cognitive Testing

A series of related cognitive tests were administered to all of the subjects using the E-prime program. The cognitive tests assessed various domains, including attention, executive function and working memory. The Attention Network Task (ANT) was used to measure the efficiency of the alerting, orienting and executive control networks to examine the interactions among these networks (Fan et al., 2006) based on the discrepancies in reaction time (RT) with different types of cues and flanker stimuli. Executive function was evaluated with the Stroop Test (Liu et al., 2017), and the one-back working memory task was used to evaluate the subjects’ memory (Wang et al., 2011). The results of the Stroop Test and the one-back Working Memory Test were assessed based on the accuracy of the answers and the mean RT during the task.

### Image Preprocessing

Data Processing Assistant for Resting-State Brain Imaging (DPABI^1^) Advanced Edition software was used to conduct the functional image processing on the MATLAB 8.2.0.701 (R2013b) platform. The following preprocessing steps were used. The functional images that were collected at the first 10 time points were discarded to eliminate the influence of subject adjustment to the new and noisy environment: (i) Slice timing: the remaining 260 volumes of functional images were corrected for temporal shifts between slices; (ii) Realignment: we excluded the subjects whose head motion was more than 1.5 mm in any direction or whose head rotation was greater than 1.5° in any angular dimension; (iii) Coregistration: the structural images were coregistered to the functional images; (iv) Segmentation: the whole brain was divided into gray matter (GM), white matter (WM) and cerebrospinal fluid (CSF), and this information was used in the subsequent spatial normalization; (v) Nuisance covariate regression: the Friston 24-parameter model, including six head motion parameters, six head motion parameters one time point before and the 12 corresponding squared items, was chosen as a strict method to reduce noise. Furthermore, noise from the CSF and WM was also regressed, and head motion scrubbing was performed with a threshold of approximately 0.5 mm. Notably, although there is controversy over whether to remove global brain signals, we retained the global signals (Liu et al., 2017); (vi) Normalize: the GM was spatially normalized to the standard Montreal Neurological Institute (MNI) template and each voxel size was resampled to 3 mm × 3 mm × 3 mm; (vii) Smooth: a 6-mm full width at half-maximum (FWHM) Gaussian kernel was used to conduct spatial smoothing; (viii) Filter: bandpass filtering was conducted to decrease low-frequency drift (*f* < 0.01 Hz) and high-frequency physiological noise (*f* > 0.08 Hz). In addition, detrending was used to remove the linear trends.

### DC and FC Calculations

DC calculations were performed to locate the impaired hubs that had altered connections with the other voxels across the whole brain at the voxel level, using the preprocessed fMRI data to calculate the voxel-based whole-brain functional correlation to acquire the voxel wise DC. Pearson’s correlation coefficient (*r*) between each pair of brain GM voxels was calculated. Thus, we obtained a matrix consisting of Pearson correlation coefficients describing the whole-brain FC model. To obtain a graph for each subject, we constructed a functional network of the whole brain by defining a threshold for each correlation at *r* > 0.25. This threshold was chosen to remove voxels that had low temporal correlation caused by noise (Göttlich et al., 2015). The FC analysis was conducted on the MATLAB platform with an rs-fMRI data analysis toolkit (Song et al., 2011; REST v1.8^2^). The peak points from the results of the DC analysis were defined as the coordinates of the seed regions, and the radius of each seed was 6 mm. Once the seeds were defined, seed-based FC analyses were carried out, and Pearson’s correlation analyses were conducted between the seeds and the remaining brain voxels. Finally, Fisher’s *r*-to-*z* transformation was applied to all maps of DC and FC before statistical analysis.

### Statistical Analysis

Independent samples *t*-tests were performed in SPSS 22.0 to analyze the differences in demographics, neuropsychological ANT data, Stroop RT and one-back RT between the two groups. Simultaneously, the chi-squared test was performed to detect differences in the accuracy of the Stroop and one-back tasks. Statistical significance was defined as *p* < 0.05.

Two-sample *t*-tests were applied in SPM8^3^ software to investigate the clear differences in the statistical maps of DC and FC between early postmenopausal women and premenopausal controls. Age and educational level were used as covariates in the calculation. A rigid approach, known as the topological false discovery rate (FDR), was used to correct for multiple comparisons (Chumbley et al., 2010). We set the voxel-level threshold at *p* = 0.001 and the FDR statistical significance at *p* < 0.05, and the FDR correction was applied to multiple comparisons across the whole brain. In addition, small-volume correction (SVC) was applied over the following brain regions predicted a priori to show abnormalities in postmenopausal women based on recent structural and functional MRI studies on menopause (Maki et al., 2011; De Bondt et al., 2015; Chhibber et al., 2017): occipital gyrus, insula, frontal cortex, temporal gyrus, amygdala, hippocampus/parahippocampal gyrus, cingulate cortex. SVC correction was conducted by applying a familywise error (FWE)-corrected threshold of *p* < 0.05 over the volume of the SVC based region, and the volume of the brain region was required to fulfill a cluster size threshold of 20 contiguous voxels. SVC is a hypothesis-driven analytical approach to multiple comparisons in specific ROIs and serves as an alternative to other corrections for the whole brain (Torres et al., 2016).

To investigate the relationships among all of the neuropsychological results, clinical symptoms, the mean *z* value extract from the significantly changed brain areas in early postmenopausal women, we conducted Pearson correlation analyses with SPSS software after eliminating the influences of age and education level and the statistical significance threshold was set at *p* < 0.05.

## Results

### Demographic and Neuropsychological Results

The demographic and neuropsychological results for the early postmenopausal women and premenopausal controls are presented in Table 1. There were no statistically significant differences in age (*p* = 0.252) or educational level (*p* = 0.459). Compared with the control group, the early postmenopausal group obtained significantly higher scores on the KI (*p* = 0.002), BDI-II (*p* < 0.001) and PSQI (*p* < 0.001), and the neuropsychological results showed that the postmenopausal group had longer alerting times (*p* = 0.033), executive control times (*p* = 0.010), one-back working memory RTs (*p* = 0.024) and Stroop Test RTs (*p* = 0.003) but lower accuracy rates in the one-back Working Memory Test (*p* < 0.001) and Stroop Test (*p* < 0.001). The postmenopausal group had significantly higher serum FSH levels (*p* < 0.001) and lower estrogen levels than the control group.

**Table 1:** Demographic, clinical characteristics, mood and cognitive performance data.

Characteristic	Postmenopausal women	Premenopausal women	*p*-value
*n*	43	44	
Age (years)	47.37 ± 1.59	46.98 ± 1.61	0.252^a^
Edu (years)	13.60 ± 1.51	13.36 ± 1.51	0.459^a^
FSH	52.82 ± 24.86	4.87 ± 3.26	<0.001^a^
KI	13.19 ± 7.41	8.59 ± 5.58	0.002^a^
BDI-II	18.09 ± 7.05	7.91 ± 8.29	<0.001^a^
SAS	41.35 ± 9.01	41.39 ± 7.51	0.744^a^
PSQI	9.00 ± 3.64	5.00 ± 3.33	<0.001^a^
ANT			
Alerting (ms)	48.12 ± 22.79	37.74 ± 21.94	0.033^a^
Orienting (ms)	40.03 ± 22.03	34.84 ± 21.87	0.273^a^
Executive control (ms)	128.24 ± 36.69	109.39 ± 32.10	0.010^a^
One-Back			
ACC	0.9149 ± 0.0544	0.9525 ± 0.0227	<0.001^b^
RT (ms)	1096.98 ± 312.48	969.07 ± 192.16	0.024^a^
Stroop Test			
ACC	0.8407 ± 0.1079	0.9266 ± 0.0913	<0.001^b^
RT (ms)	836.04 ± 127.36	763.94 ± 85.78	0.003^a^

*Abbreviations: Edu, education; FSH, follicle-stimulating hormone; KI. Kupperman index; BDI-II, Beck depression inventory II; SAS, self-rating anxiety scale; PSQI, Pittsburgh Sleep Quality Index; ANT, Attention Network Task; ACC, accuracy rating; RT, reaction time. ^a^Two-sample independent *t*-tests (43 early postmenopausal women vs. 44 controls). ^b^Chi-square test (43 early postmenopausal women vs. 44 controls)*.

### DC Analysis

**Figure 1:**
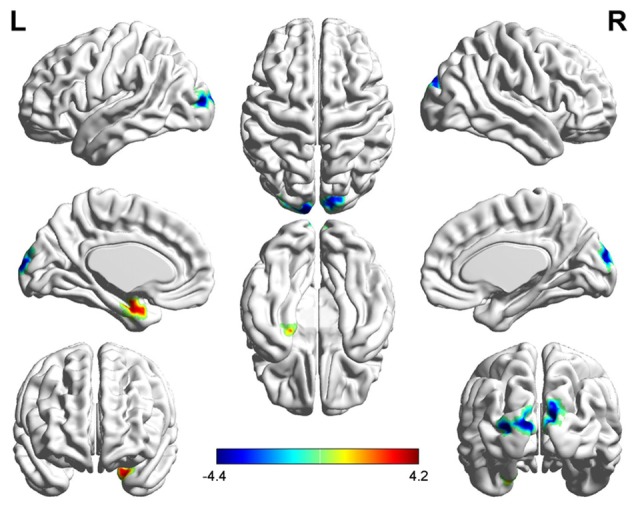
Significantly increased (red) and decreased (blue) degree centrality (DC) in early postmenopausal women compared with controls (the peak level threshold was set at *p* = 0.001; cluster level false discovery rate (FDR)-corrected *p* < 0.05 or Small-volume corrected, familywise error (FWE)-corrected at *p* < 0.05 with a cluster size threshold of 20 contiguous voxels). The color bar indicates the *t*-value from the two-sample *t*-test between the two groups.

Three related brain regions exhibited significant DC alterations in the early postmenopausal women compared with the premenopausal controls (*p* < 0.05, FDR-corrected, voxel-level threshold of *p* = 0.001 or Small-volume corrected, FWE-corrected at *p* < 0.05 with a cluster size threshold of 20 contiguous voxels; Figure 1, Table 2). Early postmenopausal women had increased DC values with a peak difference in the left amygdala (AMYG.L) and decreased DC values with the peak differences in the left middle occipital gyrus (MOG.L) and right middle occipital gyrus (MOG.R). The abovementioned seed regions were used for subsequent FC analyses.

**Table 2:** DC alterations between the two groups.

		MNI peak point coordinates				
Brain region	BA	*X*	*Y*	*Z*	*t*-value	Voxels
AMYG.L^b^	35	−24	−3	−27	4.0298	45
MOG.L^a^	18	−12	−99	12	−4.963	61
MOG.R^b^	19	9	−96	12	−4.1971	37

*Notes: MNI, Montreal Neurological Institute space; DC, degree centrality; BA, Brodmann’s area; AMYG.L, left amygdala; MOG.L, left middle occipital gyrus; MOG.R, right middle occipital gyrus. ^a^False discovery rate (FDR) corrected, *p* < 0.05. ^b^Small-volume corrected (familywise error (FWE)-corrected at p < 0.05) with a cluster size threshold of 20 contiguous voxels*.

### Seed-Based FC Analysis

The AMYG.L, MOG.L and MOG.R were set as seed points, and two-sample *t*-tests were applied to the statistical maps obtained with the seed-based analysis between the postmenopausal group and the control group to detect significantly altered FC between the seed points and the whole brain (Table 3).

**Table 3:** FC alterations between the two groups.

			MNI coordinates				
Connected regions	BA	Peak areas	*X*	*Y*	*Z*	*t*-value	Voxels
Seed point (−24, −3, −27)							
	13	INS.L^b^	−30	−33	9	3.7447	38
	9/10	Bilateral PFC^b^	0	54	33	3.9359	39
	10	SFG.R^b^	3	−6	78	4.1754	78
	17	IOG.L^b^	−18	−99	−15	3.6212	29
Seed point (−12, −99, 12)
	19	MOG.L^a^	−21	−72	0	−4.6295	196
	18	MOG.R^a^	18	−75	9	−4.59	183
	10	SFG.R^b^	21	63	18	−4.3991	25
	2	IPG.L^a^	−39	−30	36	−3.9269	122
	6	MFG.L^b^	−33	3	54	−3.8833	51
	5	SFG.R^b^	6	−42	63	−3.6806	40
Seed point (9, −96, 12)							
	20	MTG.R^a^	60	−45	−24	−4.1129	149
	47	IFG.R^b^	42	30	−21	−4.0295	44
	17	MOG.L^a^	−18	−102	−3	−4.0609	168
	18	MOG.R^b^	18	−102	−9	−3.7911	76
	10	SFG.R^a^	18	57	21	−4.2723	127
	2	IPG.L^b^	−39	−30	36	−3.8902	70
	9	MFG.R^b^	36	18	42	−4.167	44
	7	SPG.L^b^	−30	−57	66	−4.299	77

*Notes: FC, functional connectivity; INS.L, left insula; bilateral PFC, bilateral prefrontal cortex; SFG.R, right superior frontal gyrus; IOG.L, left inferior occipital gyrus; IPG.L, left inferior parietal gyrus; MFG.L, left middle frontal gyrus; MTG.R, right middle temporal gyrus; IFG.R, right inferior frontal gyrus; MFG.R, right middle frontal gyrus; SPG.L, left superior parietal gyrus; FEW, familywise error. ^a^FDR-corrected, *p* < 0.05. ^b^Small-volume corrected (FWE-corrected at p < 0.05) with a cluster size threshold of 20 contiguous voxels*.

**Figure 2:**
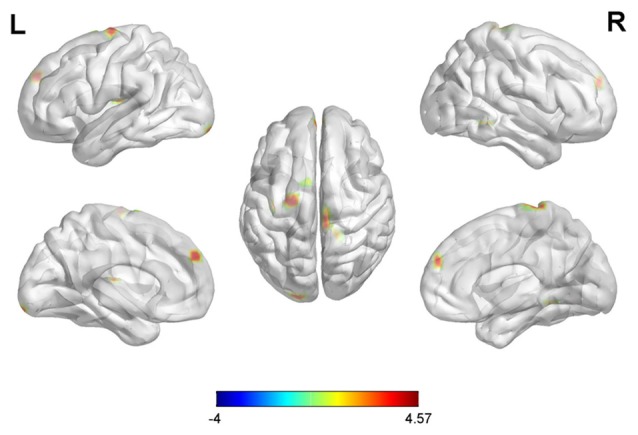
Differences in left amygdala (AMYG.L) based functional connectivity (FC) between early postmenopausal women and controls (the peak level threshold was set at *p* = 0.001, small-volume correction (SVC)-corrected; FWE-corrected at *p* < 0.05). All the FC strengths were increased and marked in red.

With the peak point (−24, −3, −27) of the AMYG.L as the first seed point, significantly increased FC strength was shown in some brain regions, including the left insula (INS.L), bilateral prefrontal cortex (PFC), left inferior occipital gyrus (IOG.L) and right superior frontal gyrus (SFG.R; SVC corrected; Figure 2).

**Figure 3:**
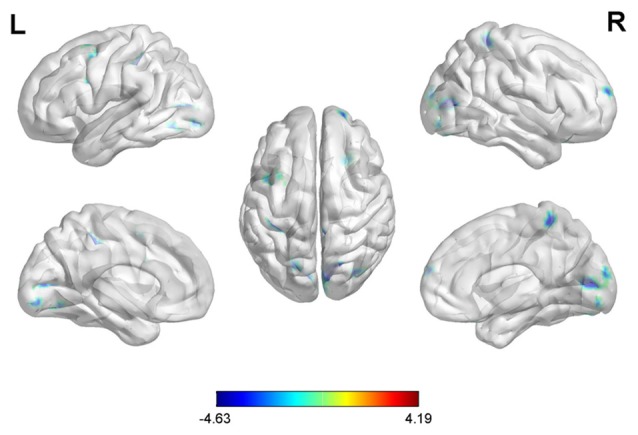
Differences in left middle occipital gyrus (MOG.L) based FC between early postmenopausal women and controls (the peak level threshold was set at *p* = 0.001; cluster level FDR-corrected *p* < 0.05 or Small-volume corrected, FWE-corrected at *p* < 0.05 with a cluster size threshold of 20 contiguous voxels). All the FC strengths were decreased and marked in blue.

With the peak point (−12, −99, 12) of the MOG.L as the second seed point, decreased FC strength was revealed in some brain regions, including the SFG.R, left inferior parietal gyrus (IPG.L), left middle frontal gyrus (MFG.L) and right superior parietal gyrus (SPG.R; *p* < 0.05, FDR corrected or SVC corrected; Figure 3).

**Figure 4:**
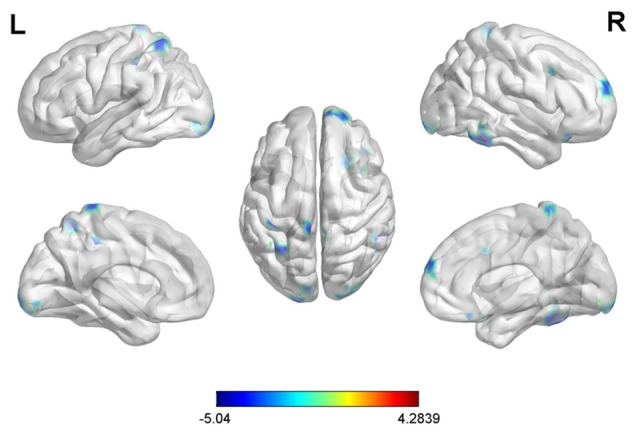
Differences in right middle occipital gyrus (MOG.R) based FC between early postmenopausal women and controls (the peak level threshold was set at *p* = 0.001; cluster level FDR-corrected *p* < 0.05 or Small-volume corrected, FWE-corrected at *p* < 0.05 with a cluster size threshold of 20 contiguous voxels). All the FC strengths were decreased and marked in blue.

With the peak point (9, −96, 12) of the MOG.R as the third seed point, decreased FC strength was revealed in some brain regions, including the right middle temporal gyrus (MTG.R), right inferior frontal gyrus (IFG.R), SFG.R, IPG.L, right middle frontal gyrus (MFG.R) and left superior parietal gyrus (SPG.L; *p* < 0.05, FDR corrected or SVC corrected; Figure 4).

### Correlation Among Neuropsychological Data, Abnormal DC Values and FC Values

**Figure 5:**
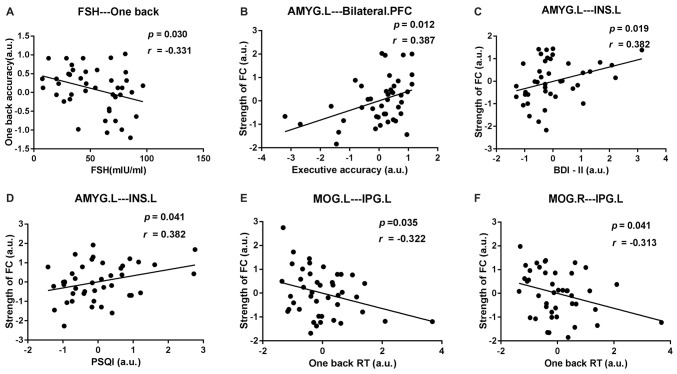
Scatter diagrams showing the significant pair-wise correlations between abnormal DC, rsFC, clinical and neuropsychological data in early postmenopausal women in **(A–F)**. **(A)** The serum follicle-stimulating hormone (FSH) level was negatively correlated with the working memory accuracy rate. **(B)** The executive function accuracy rate was positively correlated with the increased FC strength between the AMYG.L and bilateral prefrontal cortex (PFC). **(C,D)** The increased FC strength between the AMYG.L and left insula (INS.L) was simultaneously positively correlated with the BDI-II and pittsburgh sleep quality index (PSQI) score. **(E)** The working memory reaction time (RT) was negatively correlated with the decreased FC strength between the MOG.L and left inferior parietal gyrus (IPG.L.) **(F)** The working memory RT was negatively correlated with the decreased FC strength between the MOG.R and IPG.L.

After age and educational level were regressed out, Pearson correlation analyses were conducted among the early postmenopausal women. First, the serum FSH level was negatively correlated with the working memory accuracy rate (*p* = 0.030, *r* = −0.331). Subsequently, the executive function accuracy rate was positively correlated with the increased FC strength between the AMYG.L and bilateral PFC (*p* = 0.012, *r* = 0.387). In addition, the increased FC strength between the AMYG.L and INS.L was positively correlated with the BDI-II and PSQI scores (*p* = 0.019, *r* = 0.382; *p* = 0.041, *r* = 0.382, respectively). Finally, the working memory RT was negatively correlated with the decreased FC strength between the MOG.L and IPG.L (*p* = 0.035, *r* = −0.322) also negatively correlated with the decreased FC strength between the MOG.R and IPG.L (*p* = 0.041, *r* = −0.313). The abovementioned outcomes are shown in Figure 5.

## Discussion

This was the first study to investigate the aberrant cerebral activities between the early postmenopausal women and the age-matched controls using the DC and FC approaches. Three observations were made: (1) increased DC values in the AMYG.L and decreased DC values in bilateral MOG in early postmenopausal women; (2) further seed-based FC analyses showed increased FC between the AMYG.L and other brain areas, such as the bilateral PFC, INS.L, IOG.L and SFG.R; decreased FC from the MOG.L to other regions, including the SFG.R, SPG.R, MFG.L and IPG.L; decreased FC from the MOG.R to other regions, including the MTG.R, IFG.R, SFG.R, IPG.L, MFG.R and SPG.L; and (3) Pearson correlation analysis revealed that the increased FC strength between the AMYG.L and bilateral PFC was associated with executive function; the value of DC in AMYG.L was also positively correlated with the executive function; the increased FC between the AMYG.L and INS.L was simultaneously related to the BDI-II and PSQI scores; bilateral MOG have the decreased FC strength with the IPG.L which was connected to working memory. As discussed below, the results of this study illuminated the potential mechanisms underlying the cognitive impairments and neuropsychological complications during the postmenopausal period.

The altered DC values might be related to hormone disorders in early postmenopausal subjects. Estrogen has neuroprotective effects, and the mechanisms of these effects have been well documented in neurodegenerative disorders and neuroinflammation by previous fundamental studies in regard to the following four aspects: (1) estrogen acts on tau protein, which is specifically distributed in some cognition-related brain regions (Ising et al., 2015); (2) estrogen is involved in the regulation of microRNAs in both neuroprotection and neurodegeneration in the CNS (Reddy et al., 2017); (3) estrogen acts on glial cells, which can express estrogen receptors and mediate neuroprotection due to estrogen in different pathologies (Arevalo et al., 2010; Barreto, 2016); and (4) decreased estrogen leads to hypometabolism in the brains of postmenopausal women by modulating mitochondrial bioenergetics (Rasgon et al., 2005; Hu et al., 2017). Based on the above-mentioned researches, the brain tissue might be damaged due to the decreased estrogen level. In this study, early postmenopausal women exhibited significantly higher DC values in the AMYG.L and lower DC values in bilateral MOG. The DC value could mirror the amount of communication with the whole-brain networks. The decreased DC value in bilateral MOG coincided with the reduced neuroprotective effects (Ising et al., 2015). The DC value in the AMYG.L was increased, first, because the function of the amygdala was activated in the early postmenopausal women who showed the distinct state of depression and anxiety in this study. Inherent harmful factors due to low levels of estrogen were present, consistent with a previous study that revealed abnormalities in the GM volumes in the amygdala in postmenopausal women (Erickson et al., 2010); moreover, this area was activated by the extensive FC from the AMYG.L to other regions including bilateral PFC, INS.L, IOG.L and SFG.R which were showed in this study.

Furthermore, the abnormal FC between the AMYG.L and bilateral PFC might be involved in the neuropathological mechanisms underlying executive function impairments. In this study, Pearson’s correlation analysis showed that the increased FC between the AMYG.L and bilateral PFC was positively correlated with executive function accuracy in the early postmenopausal women, this result was consistent to numerous prior studies: the frontal cortex plays a key role in executive function; thus, damage to the frontal cortex could lead to obvious impairments in executive function in a variety of diseases (Dreher et al., 2012; Schroeter et al., 2012); notably, a recent study demonstrated that early postmenopausal women who received HT exhibited frontal cortex activation and executive function improvements compared to others who received placebos (Girard et al., 2017). However, the early postmenopausal women had significantly decreased executive function compared to the control subjects (*p* < 0.05). Combined the two results, we can see the executive function was declined in the early postmenopausal subjects, but the subject who have a stronger FC strength will have a better executive function. Meanwhile, the estrogen acts on some specifically cognition-related brain regions (Ising et al., 2015), thus, we speculate that the enhanced FC strength in the early postmenopausal women might be responsible for the adverse effects on the brain in the early stages of hormone disorders, these effects must be based on nervous tissues, as the decreasing estrogen levels aggravate the damage to the nervous tissues and the effects of antagonism would decline, a result consistent with the so-called “critical window hypothesis” of HT in perimenopausal women (Pines, 2016).

Simultaneously, increased FC from the AMYG.L to the INS.L was shown in the early postmenopausal subjects. The insula is involved in consciousness and has a key role in diverse functions, such as negative emotional experiences and regulation of bodily homeostasis (von Leupoldt et al., 2008; Critchley, 2010). In this study, Pearson’s correlation analysis revealed that the altered FC was simultaneously positively correlated with BDI-II and PSQI scores, while the early postmenopausal women exhibited marked depression states and worse sleep quality in their daily lives compared with the controls (*p* < 0.05). Therefore, the increased FC between the AMYG.L and INS.L might be the basis of the depression and sleep disorders observed in early postmenopausal women.

Bilateral MOG and IPG probably associated with the poor memory in the early postmenopausal subjects. A previous study of AD (Golby et al., 2005) found that altered activation in the occipital gyrus was associated with damaged visual memory. De Bondt et al. (2015) also displayed that the parietal gyrus was involved in processing the number information. This study displayed decreased DC values in bilateral MOG and FC analysis showed that these two regions were simultaneously have reduced FC strength with the IPG.L. Pearson’s correlation analysis showed that the decreased FC strength between the bilateral MOG and IPG.L was negatively correlated with the working memory RT. Together, these two correlational outcomes suggested that the weaker the FC between the bilateral MOG and the IPG.L was, the worse the subject’s memory. This finding, in turn, supports the hypothesis that hormonal disorders affect cognitive function in early postmenopausal women.

There were also a few limitations to this investigation. First, the groups were composed of relatively small sample sizes (43 early postmenopausal women and 44 controls), which might have produced biased outcomes. Additionally, the social experience of these subjects was an important uncontrollable factor that may have contributed to brain function; all we could do was carefully screen the participants for similar work experience. Additionally, this experiment was carried out in a hospital; thus, the exact levels of estrogen in some of the subjects were too low to be detected, and unfortunately, this crucial hormone could not be included in the statistical or correlation analyses. Given these limitations, all these factors should be taken into consideration in future studies. In particular, as this study was a prevalence survey, a different experimental design will be necessary to test whether the alterations in brain function are reversible after HT.

## Conclusion

In conclusion, the current study revealed the dysfunction in regional and network-level brain functional relationships that might clarify the mechanisms underlying the clinical symptoms and neurobiological changes in early postmenopausal women. We found that aberrant DC and FC were mainly distributed in the AMYG.L, bilateral MOG, INS.L, bilateral PFC and IPG.L, which are associated with cognitive impairment and emotional symptoms. Thus, these findings might reveal the mechanisms underlying menopause-related brain dysfunction, and the altered patterns of DC and FC might be useful as neuroimaging biomarkers in clinical practice.

## Author Contributions

SZ contributed to performing the experiments, analyzing the data and writing the manuscript. JH and WF designed the experiment and revised the manuscript. BL, MG and CY contributed to analyzing the data and revising the manuscript. LW and GW contributed to analyzing the data and writing the manuscript. DZ is the guarantor of this study and had complete access to all the data in the study and takes responsibility for the integrity of the data and the accuracy of the data analyses.

## Conflict of Interest Statement

The authors declare that the research was conducted in the absence of any commercial or financial relationships that could be construed as a potential conflict of interest.

## Footnotes

^1^http://rfmri.org/dpabi

^2^http://www.restfmri.net/forum/REST_V1.8

^3^http://www.fil.ion.ucl.ac.uk/spm/
